# Analysis of serum B cell‐activating factor from the tumor necrosis factor family (BAFF) and its soluble receptors in systemic lupus erythematosus

**DOI:** 10.1002/cti2.1047

**Published:** 2019-04-21

**Authors:** Fabien B Vincent, Rangi Kandane‐Rathnayake, Rachel Koelmeyer, Alberta Y Hoi, James Harris, Fabienne Mackay, Eric F Morand

**Affiliations:** ^1^ Rheumatology Research Group Centre for Inflammatory Diseases School of Clinical Sciences at Monash Health Monash University Clayton VIC Australia; ^2^ Department of Immunology and Pathology Central Clinical School Alfred Medical Research and Education Precinct (AMREP) Monash University Melbourne VIC Australia; ^3^ Department of Microbiology and Immunology School of Biomedical Sciences Faculty of Medicine, Dentistry and Health Sciences The University of Melbourne Melbourne VIC Australia

**Keywords:** B cell‐activating factor from the tumor necrosis factor family, BAFF receptor, B cell maturation antigen, biomarker, systemic lupus erythematosus, transmembrane activator and cyclophilin ligand interactor

## Abstract

**Objectives:**

To determine the presence and clinical associations of the soluble receptors of B cell‐activating factor from the tumor necrosis factor family (BAFF) in serum of patients with systemic lupus erythematosus (SLE).

**Methods:**

Serum BAFF and soluble BAFF receptor (sBAFF‐R) were quantified using ELISA, and soluble B cell maturation antigen (sBCMA) and transmembrane activator and cyclophilin ligand interactor (sTACI) by Luminex, in 87 SLE patients and 17 healthy controls (HC). Disease activity and organ damage were assessed using SLE Disease Activity Index 2000 (SLEDAI‐2K) and Systemic Lupus International Collaborating Clinics (SLICC) SLE Damage Index (SDI), respectively.

**Results:**

BAFF and all receptors were detectable in all serum samples. Serum sBCMA and sTACI, but not sBAFF‐R, were significantly higher in SLE than in HC. Serum BAFF was also increased in SLE, but this association was attenuated after adjusting for age and ethnicity. Increased serum BAFF was associated with flare and organ damage. Increased serum sBCMA was associated with the presence of anti‐dsDNA, but not with overall or organ‐specific disease activity, flare or organ damage. Neither sTACI nor sBAFF‐R was associated with any SLE clinical parameters in multivariable analysis. While serum BAFF correlated negatively with sBAFF‐R in HC, no statistically significant correlations were observed between BAFF and its receptors in SLE patients.

**Conclusion:**

Serum BAFF was associated with flare and organ damage independent of the presence of its soluble receptors. While sBCMA was associated with anti‐dsDNA positivity, other soluble BAFF receptors were not associated with SLE clinical indicators.

## Introduction

Systemic lupus erythematosus (SLE) is an unpredictable and multifaceted chronic systemic autoimmune disease.^1^ One of the most prominent breakthroughs in SLE has been the discovery of the pathogenic role of B cell‐activating factor from the tumor necrosis factor (TNF) family (BAFF) [also known as B lymphocyte stimulator (BLyS)].^2^ BAFF has a crucial role in B cell maturation, differentiation and survival, and is part of the BAFF/a proliferation‐inducing ligand (APRIL) system. BAFF and APRIL ligate two cognate receptors, transmembrane activator and cyclophilin ligand interactor (TACI) and B cell maturation antigen (BCMA), and BAFF also ligates BAFF receptor (BAFF‐R).^2^ These receptors transduce distinct signals; BCMA activation is important for long‐lived plasma cell survival, BAFF‐R activation for survival and maturation of immature B cells and TACI for B cell regulation, class‐switch recombination and T cell‐independent antibody responses.^2^ BAFF‐transgenic mice develop SLE‐like features,^3^ and high serum BAFF levels are a feature of some mouse models of SLE‐like disease.^4^

Compared to healthy controls (HC), patients suffering from SLE harbour significantly higher serum BAFF levels.^2^ Serum BAFF has also been reported to be associated with disease activity and autoantibody levels in some studies.^2^ The efficacy of a BAFF‐targeting therapy, belimumab,^5^, ^6^ gives weight to the fact that BAFF plays a critical pathogenic role in SLE. Nevertheless, this therapy is effective only in a subset of SLE patients,^5^, ^6^ suggesting a potential BAFF‐mediated subset of SLE, and a significant unmet need for tools to stratify patients in order to define who may benefit from such therapy.

Published studies regarding the potential role of serum BAFF as a SLE biomarker are inconsistent,^2^ both at the overall and organ‐specific disease activity level. Amongst all potential causes for these discrepancies, the presence of soluble BAFF receptors in human SLE patients needs to be considered. Hoffmann *et al*. reported the existence of a soluble form of TACI (sTACI) as the ectodomain of transmembrane TACI, produced following cleavage of TACI by the metalloproteinase ADAM10 from the cell surface of activated B cells. When further cleaved by γ‐secretase, sTACI can oligomerise to act as a decoy receptor for both BAFF and APRIL.^7^ The same group also reported the presence of a soluble form of BCMA (sBCMA) in human sera, produced from cleavage of BCMA from plasma cells by γ‐secretase, which *in vitro* acts as a decoy receptor specific for APRIL.^8^ The same group showed that sBCMA can also be shed by plasmacytoid dendritic cells via a similar γ‐secretase‐dependent cleavage.^9^ One study has reported the existence of sBAFF‐R,^10^ a soluble form of the receptor released by human decidual stromal cells *ex vivo*, and an inhibitory role in the regulation of interleukin (IL)‐6 and TNF secretion by monocytes has been suggested.^10^ Some published studies have reported the presence of soluble forms of BAFF receptors in human serum. All three soluble BAFF receptors have been reported in rheumatoid arthritis,^11^ and sTACI and sBCMA have been described in multiple sclerosis, multiple myeloma and patients with chronic lymphocytic leukaemia.^7^, ^8^, ^12^, ^13^, ^14^ One group recently reported the presence of sBCMA and sTACI in human SLE^7^, ^8^; however, this was in a cohort of modest size (*N* < 50), and did not investigate clinical phenotypic associations, or association with flare or organ damage. To date, there are no publications on sBAFF‐R in SLE.

Here, we aimed to determine the presence and clinical associations of serum soluble BAFF receptors in SLE.

## Results

### Participants’ baseline characteristics

This study included 87 SLE patients, whose baseline characteristics are displayed in Table 1. Briefly, median [interquartile ranges (IQR)] age and disease duration were 44.3 [33.2, 56.4] and 7 [3.8, 14.8] years, respectively. The cohort was predominantly female (89%), 56% of patients were of Asian ethnicity, 36% of patients had active disease, and 62% had permanent organ damage. In all, 57% and 51% of patients were receiving glucocorticoids and immunosuppressants, respectively. Seventeen healthy individuals were enrolled in the HC group, with a median [IQR] age of 41 [28, 44] years, and comprising 88% of female and 29% of individuals of Asian ethnicity. The HC cohort was gender‐matched to the SLE cohort (Table 2). We observed a significant difference in age and a trend towards significant difference in ethnicity between SLE and HC cohorts (Table 2). Therefore, we adjusted for age and ethnicity in multivariable regression models assessing the associations of serum BAFF and soluble BAFF receptors expressions in SLE compared to HC.

**Table 1:** Demographic, clinical and biological characteristics of the SLE cohort at baseline

Characteristics	SLE cohort (*N* = 87)
Age (years), median [IQR]	44.3 [33.2, 56.4]
Female, *n* (%)	77 (89%)
Asian ethnicity, *n* (%)	49 (56%)
Disease duration (years), median [IQR]	7 [3.8, 14.8]
SLEDAI‐2K, median [IQR]	4 [2, 6]
SLEDAI‐2K > 4, *n* (%)	31 (36%)
Organ‐specific manifestations^a^	*n* (%)
Fever	0 (0%)
Neurological	1 (1%)
Renal	19 (22%)
Mucocutaneous	18 (21%)
Musculoskeletal	7 (8%)
Serosal	2 (2%)
Vascular	0 (0%)
Serological	63 (72%)
Haematological	3 (3%)
Flare^b^, *n* (%)	22 (25%)
SLICC‐SDI, median [IQR]	1 [0, 2]
SLICC‐SDI > 0, *n* (%)	54 (62%)
Treatment	*n* (%)
Prednisone	50 (57%)
Hydroxychloroquine	74 (85%)
Immunosuppressants^c^	44 (51%)
Clinical laboratory data	
CRP (mg L^−1^), median [IQR]	1.5 [0.6, 3.5]
ESR (mm h^−1^), median [IQR]	15 [8, 27]
UPCR (g mmol^−1^), median [IQR]	0.02 [0.01, 0.05]
Proteinuria^d^, *n* (%)	17 (20%)
C3 (g L^−1^), mean (SD)	0.85 (0.26)
C4 (g L^−1^), mean (SD)	0.17 (0.08)
ANA +ve (> 1280), *n* (%)	67 (81%)
Anti‐dsDNA +ve, *n* (%)	49 (56%)
Anti‐Sm Ab +ve, *n* (%)	20 (24%)

Data are expressed as mean (SD), median [IQR] or as number (percentage).

Ab, antibody; ANA, antinuclear antibody; C3, complement component 3; C4, complement component 4; CRP, C‐reactive protein; dsDNA, double‐stranded deoxyribonucleic acid; ESR, erythrocyte sedimentation rate; IQR, interquartile range; SD, standard deviation; SLE, systemic lupus erythematosus; SLEDAI‐2K, SLE Disease Activity Index 2000; SLICC‐SDI, Systemic Lupus International Collaborating Clinics‐SLE Damage Index; Sm, Smith; UPCR, urine protein/creatinine ratio.

a Individual organ domain disease activity was assessed by the SLEDAI‐2K score.

b Encompasses mild, moderate and severe flares.

c Immunosuppressants include methotrexate, azathioprine, mycophenolate mofetil, mycophenolate acid, leflunomide, cyclosporine A and/or cyclophosphamide.

d Proteinuria defined if UPCR > 0.05 g mmol^−1^.

**Table 2:** Demographics in SLE and HC

	HC (*n* = 17)	SLE (*n* = 87)	*P*‐value
Demographics			
Age (years), median [IQR]	41 [28, 44]	44.3 [33.2, 56.4]	0.04
Female, *n* (%)	15 (88%)	77 (89%)	0.9
Asian, *n* (%)	5 (29%)	49 (56%)	0.06

Data are expressed as median [IQR] or as number (percentage).

HC, healthy control; IQR, interquartile range; SLE, systemic lupus erythematosus.

### Serum BAFF and soluble BAFF receptors concentrations in SLE

**Figure 1:**
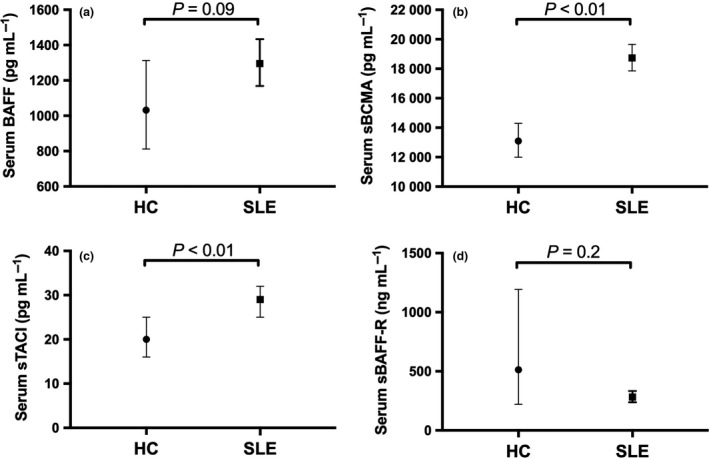
Serum BAFF and soluble BAFF receptors in SLE and HC. **(a)** Geometric mean (GM) of serum BAFF concentrations in HC (N = 17) and SLE (N = 87). **(b)** GM of serum sBCMA concentrations in HC (N = 17) and SLE (N = 87). **(c)** GM of serum sTACI concentrations in HC (N = 17) and SLE (N = 87). **(d)** GM of serum sBAFF‐R concentrations in HC (N = 11) and SLE (N = 87). In **a–c**, horizontal bars indicate the age‐ and ethnicity‐adjusted GM (95% CI) derived from multivariable linear regression analyses. In **d**, horizontal bars indicate the GM (95% CI) derived from univariable linear regression analyses.

**Figure 2:**
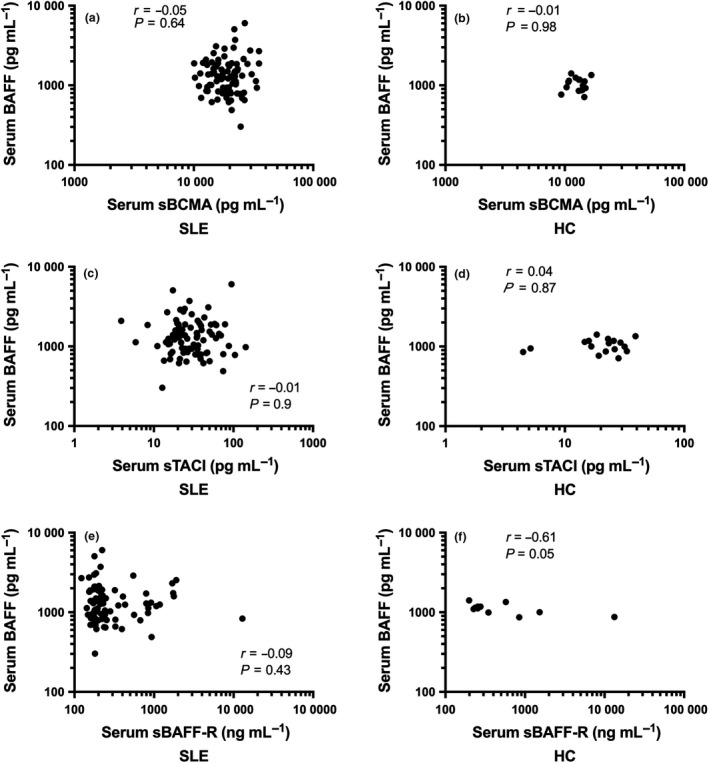
Correlation between serum BAFF and soluble BAFF receptors in SLE and HC. Correlation between serum BAFF and sBCMA concentrations in **(a)** SLE (N = 87) and **(b)** HC (N = 17). Correlation between serum BAFF and sTACI concentrations in **(c)** SLE (N = 87) and **(d)** HC (N = 17). Correlation between serum BAFF and sBAFF‐R concentrations in **(e)** SLE (N = 87) and **(f)** HC (N = 11). In **a**–**f**, correlations were examined using Spearman correlation test.

BAFF was detectable in all serum samples from SLE patients and HC. Univariable linear regression analysis showed an association of increased serum BAFF levels in SLE compared to HC of borderline significance (ratio of geometric mean (GM), 1.27; 95% CI 0.99, 1.63; *P *=* *0.06). However, this association was weakened after adjusting for age and ethnicity (ratio of GM 1.25; 95% CI 0.96, 1.64; *P *=* *0.09; Figure 1a; Supplementary table 1). sBCMA, sTACI and sBAFF‐R were also detectable in all SLE and HC serum samples. There was evidence of increased serum sBCMA and sTACI in the SLE group (sBCMA: ratio of GM 1.46; 95% CI 1.35, 1.58; *P *<* *0.01; and sTACI: ratio of GM 1.47; 95% CI 1.11, 1.94; *P *<* *0.01), confirmed after adjusting for age and ethnicity (sBCMA: ratio of GM 1.43; 95% CI 1.28, 1.59; *P *<* *0.01; sTACI: ratio of GM 1.45; 95% CI 1.11, 1.91; *P *<* *0.01; Figure 1b, c; Supplementary tables 2, 3). Serum BAFF was not significantly correlated with concentrations of any soluble BAFF receptors in SLE (Figure 2). We did not find any significant difference in serum sBAFF‐R levels between SLE and HC (Figure 1d). However, serum BAFF was significantly negatively correlated with serum sBAFF‐R, while not with sBCMA and sTACI, in HC subjects but not in SLE (Figure 2).

### Serum BAFF and SLE clinical parameters

**Figure 3:**
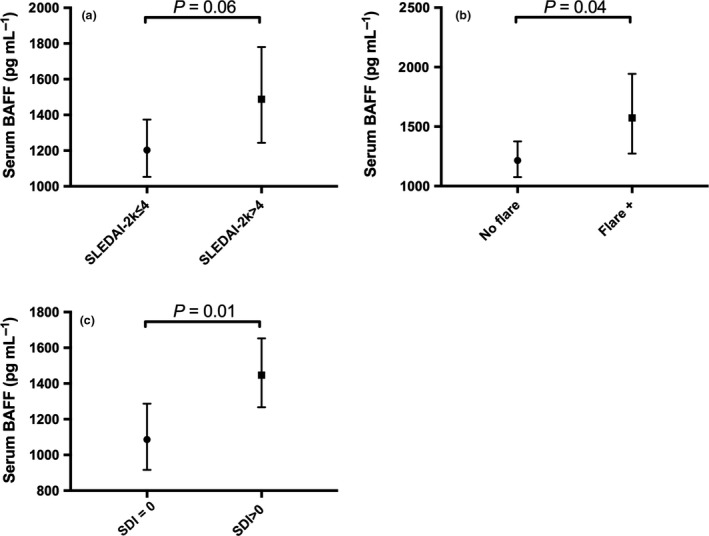
Associations of serum BAFF with SLE clinical parameters. **(a)** Geometric mean (GM) of serum BAFF concentrations in SLE patients with inactive disease (SLEDAI‐2K ≤ 4: *n* = 56) versus patients with active disease (SLEDAI‐2K > 4: *n* = 31). **(b)** GM of serum BAFF concentrations in SLE patients with no flare (*n* = 65) versus patients with flare of disease (*n* = 22). **(c)** GM of serum BAFF concentrations in SLE patients with no organ damage (SDI = 0: *n* = 33) versus patients with organ damage (SDI > 0: *n* = 54). In **a–c**, horizontal bars indicate the GM (95% CI) derived from univariable linear regression analyses.

We next evaluated potential associations between serum BAFF with demographics and clinical parameters using linear regression. Univariable analysis revealed associations between increased serum BAFF and active disease, flare and organ damage. Serum BAFF levels were greater in SLE patients with active disease with borderline significance (ratio of GM 1.24; 95% CI 0.99, 1.55; *P *=* *0.06; Figure 3a), flare of disease (ratio of GM 1.29; 95% CI 1.01, 1.65; *P *=* *0.04; Figure 3b) or irreversible organ damage compared to those without (ratio of GM 1.33; 95% CI 1.07, 1.65; *P *=* *0.01; Figure 3c, Table 3). The association between increased serum BAFF and organ damage was confirmed in multivariable analysis, after adjusting for disease duration (adjusted ratio of GM 1.29; 95% CI 1.03, 1.6; *P *=* *0.02). However, the association between increased serum BAFF and active disease was attenuated after adjusting for the use of immunosuppressants (adjusted ratio of GM 1.18; 95% CI 0.93, 1.48; *P *=* *0.17). With respect to laboratory markers, increased serum BAFF concentrations were significantly associated with high ESR, but not with other routine laboratory markers (Table 3). Serum BAFF was significantly lower in patients receiving hydroxychloroquine, while significantly higher in those receiving immunosuppressants (Table 3). No significant association was observed between serum BAFF concentrations and other clinical parameters (Table 3).

**Table 3:** Univariable associations of serum BAFF and its soluble receptors in SLE at baseline

	SLE cohort (N = 87)							
	BAFF		sBAFF Receptors					
			sBCMA		sTACI		sBAFF‐R	
	RC (95% CI)	*P*‐value	RC (95% CI)	*P*‐value	RC (95% CI)	*P*‐value	RC (95% CI)	*P*‐value
Demographics								
Age	1.00 (0.99, 1.01)	0.49	1.00 (0.99, 1.01)	0.14	1.00 (0.99, 1.01)	0.97	0.98 (0.97, 1.00)	0.02
Disease duration	1.01 (1.00, 1.03)	0.05	1.01 (0.99, 1.01)	0.11	1.00 (0.98, 1.02)	0.78	0.98 (0.96, 0.99)	<0.01

	Ratio of GM (95% CI)	*P*‐value	Ratio of GM (95% CI)	*P*‐value	Ratio of GM (95% CI)	*P*‐value	Ratio of GM (95% CI)	*P*‐value
Demographics								
Male	1.11 (0.79, 1.57)	0.53	1.02 (0.86, 1.21)	0.86	1.29 (0.96, 1.72)	0.09	1.34 (0.7, 2.53)	0.38
Asian ethnicity	0.93 (0.75, 1.16)	0.52	0.95 (0.82, 1.09)	0.45	0.98 (0.75, 1.29)	0.91	1.42 (1.01, 2.02)	0.04
Clinical manifestations								
Disease activity (SLEDAI‐2K)								
Overall	1.24 (0.99, 1.55)	0.06	0.93 (0.82, 1.05)	0.26	0.83 (0.66, 1.05)	0.12	1.04 (0.74, 1.47)	0.82
Serological	1.16 (0.91, 1.48)	0.22	1.06 (0.94, 1.19)	0.36	0.89 (0.71, 1.11)	0.31	1.35 (1.02, 1.78)	0.04
Renal	1.19 (0.92, 1.55)	0.19	0.92 (0.77, 1.09)	0.33	1.16 (0.89, 1.52)	0.26	1.04 (0.72, 1.51)	0.82
Mucocutaneous	0.99 (0.75, 1.29)	0.93	0.99 (0.87, 1.13)	0.92	0.82 (0.62, 1.09)	0.17	1.11 (0.77, 1.59)	0.58
Mild/moderate flare	1.26 (0.95, 1.68)	0.11	0.98 (0.82, 1.16)	0.81	0.91 (0.72, 1.15)	0.44	1.05 (0.68, 1.62)	0.83
Severe flare	1.17 (0.82, 1.68)	0.37	1.12 (0.79, 1.59)	0.53	1.24 (0.85, 1.8)	0.27	0.83 (0.59, 1.17)	0.3
Flare any	1.29 (1.01, 1.65)	0.04	1.06 (0.88, 1.27)	0.56	1.08 (0.82, 1.41)	0.59	0.99 (0.67, 1.48)	0.98
Organ damage present (SDI > 0)	1.33 (1.07, 1.65)	0.01	0.98 (0.88, 1.09)	0.69	1.05 (0.8, 1.4)	0.71	1.04 (0.74, 1.47)	0.83
Treatment at baseline								
Prednisolone	0.98 (0.79, 1.22)	0.86	0.98 (0.87, 1.11)	0.78	0.82 (0.62, 1.08)	0.15	1.06 (0.8, 1.4)	0.68
Hydroxychloroquine	0.65 (0.49, 0.88)	<0.01	0.99 (0.79, 1.26)	0.96	0.87 (0.49, 1.56)	0.65	1.41 (1.04, 1.91)	0.03
Immunosuppressants^a^	1.23 (0.996, 1.53)	0.05	0.91 (0.81, 1.03)	0.13	0.85 (0.68, 1.07)	0.17	1.22 (0.9, 1.66)	0.2
Laboratory markers								
High CRP (> 3)	1.23 (0.96, 1.57)	0.096	0.99 (0.84, 1.17)	0.95	1.23 (0.9, 1.69)	0.18	0.9 (0.56, 1.47)	0.68
High ESR (≥ 25)	1.53 (1.23, 1.9)	<0.01	1.05 (0.92, 1.19)	0.48	0.94 (0.75, 1.19)	0.62	1.29 (0.77, 2.15)	0.33
Proteinuria (UPCR > 0.05)	1.16 (0.88, 1.52)	0.3	0.95 (0.82, 1.09)	0.45	1.15 (0.82, 1.61)	0.41	0.93 (0.64, 1.36)	0.71
Low C3 (< 0.79)	1.21 (0.97, 1.51)	0.09	1.07 (0.93, 1.22)	0.34	1.04 (0.79, 1.37)	0.78	1.03 (0.73, 1.47)	0.85
Low C4 (< 0.16)	1.06 (0.85, 1.32)	0.6	1.03 (0.91, 1.16)	0.64	0.82 (0.63, 1.07)	0.14	1.08 (0.77, 1.51)	0.64
ANA +ve (≥ 1280)	1.3 (0.98, 1.71)	0.07	1.13 (0.99, 1.29)	0.08	0.9 (0.67, 1.22	0.51	0.93 (0.57, 1.52)	0.76
Anti‐dsDNA +ve	1.07 (0.86, 1.34)	0.52	1.12 (1.00, 1.26)	0.05	1.01 (0.82, 1.24)	0.92	1.08 (0.82, 1.44)	0.58
Anti‐Sm Ab +ve	0.86 (0.66, 1.12)	0.25	1.11 (0.96, 1.27)	0.17	1.04 (0.79, 1.36)	0.8	0.84 (0.6, 1.17)	0.31
Serum cytokine and soluble receptors								
BAFF	–		1.00 (1.00, 1.00)	0.68	1.00 (1.00, 1.00)	0.79	1.00 (1.00, 1.00)	0.51
sBCMA	1.00 (1.00, 1.00)	0.87	–	–	1.00 (1.00, 1.00)	<0.01	1.00 (1.00, 1.00)	0.24
sTACI	1.00 (0.995, 1.01)	0.98	1.00 (1.00, 1.01)	<0.01	–	–	1.00 (0.99, 1.01)	0.94
sBAFF‐R	1.00 (1.00,1.00)	0.53	1.00 (1.00, 1.00)	0.8	1.00 (1.00, 1.00)	0.92	–	–

Ab, antibody; ANA, antinuclear antibody; BAFF, B cell‐activating factor from the tumor necrosis factor family; BAFF‐R, BAFF receptor; BCMA, B cell maturation antigen; C3, complement component 3; C4, complement component 4; CI, confidence interval; CRP, C‐reactive protein; dsDNA, double‐stranded deoxyribonucleic acid; ESR, erythrocyte sedimentation rate; GM, geometric mean; RC, regression coefficient; SLE, systemic lupus erythematosus; SLEDAI‐2K, SLE Disease Activity Index 2000; SLICC‐SDI, Systemic Lupus International Collaborating Clinics‐SLE Damage Index; Sm, Smith; TACI, transmembrane activator and cyclophilin ligand interactor; UPCR, urine protein/creatinine ratio.

a Immunosuppressants include methotrexate, azathioprine, mycophenolate mofetil, mycophenolate acid, leflunomide, cyclosporine A and/or cyclophosphamide.

We further characterised baseline serum BAFF in relation with longitudinally collected clinical parameters over a median length of follow‐up of 2 years following baseline assessment (Table 4). Univariable logistic regression analysis revealed an association between baseline serum BAFF and time‐adjusted mean SLEDAI‐2K (AMS), where patients who had high BAFF levels (>median) at baseline visit were more than twice likely to have AMS > 4 (OR, 2.67; 95% CI 1.1, 6.47; *P *=* *0.03; Table 5); this association, however, attenuated after adjusting for the use of immunosuppressants (adjusted OR, 2.13, 95% CI 0.84, 5.42; *P *=* *0.11). In addition, patients with high baseline BAFF levels were nearly four times more likely to have organ damage at the final visit when compared to patients with low serum BAFF levels (OR, 3.7; 95% CI 1.39, 9.81; *P *<* *0.01; Table 5). We did not find an association between BAFF and flare over time (Table 5).

**Table 4:** Clinical characteristics of the SLE cohort over time

Characteristics	SLE cohort (*N* = 87)
Length of follow‐up (years), median [IQR]	2 [1.8, 2.1]
AMS, median [IQR]	3.7 [1.7, 5.6]
AMS > 4, *n* (%)	38 (45%)
Change in SLICC‐SDI > 0, *n* (%)	19 (22%)
Flare overtime^a^, *n* (%)	59 (68%)

Data are expressed as median [IQR] or as number (percentage).

AMS, adjusted mean SLE Disease Activity Index 2000; IQR, interquartile range; SLE, systemic lupus erythematosus; SLICC‐SDI, Systemic Lupus International Collaborating Clinics‐SLE Damage Index; SLE, Systemic lupus erythematosus.

a Encompasses mild, moderate and severe flares.

**Table 5:** Univariable associations of baseline serum BAFF and its soluble receptors with SLE clinical parameters overtime

	AMS > 4		Flare overtime^a^		Organ damage present at last visit		Damage accrual	
	OR (95% CI)	*P*‐value	OR (95% CI)	*P*‐value	OR (95% CI)	*P*‐value	OR (95% CI)	*P*‐value
Baseline serum BAFF								
Low (≤ median)	1.00		1.00		1.00		1.00	
High (> median)	2.67 (1.1, 6.47)	0.03	1.83 (0.73, 4.57)	0.2	3.7 (1.39, 9.81)	<0.01	2.06 (0.72, 5.88)	0.18
Baseline serum sBCMA								
Low (≤ median)	1.00		1.00		1.00		1.00	
High (> median)	0.46 (0.19, 1.1)	0.08	0.44 (0.17, 1.11)	0.08	0.54 (0.22, 1.36)	0.19	0.64 (0.23, 1.81)	0.4
Baseline serum sTACI								
Low (≤ median)	1.00		1.00		1.00		1.00	
High (> median)	0.68 (0.29, 1.61)	0.38	1.19 (0.48, 2.94)	0.7	0.84 (0.34, 2.07)	0.7	0.64 (0.23, 1.81)	0.4
Baseline serum sBAFF‐R								
Low (≤ median)	1.00		1.00		1.00		1.00	
High (> median)	1.32 (0.56, 3.13)	0.53	1.83 (0.73, 4.57)	0.2	0.97 (0.39, 2.39)	0.94	0.68 (0.24, 1.92)	0.47

AMS, adjusted mean SLE Disease Activity Index 2000; BAFF, B cell‐activating factor from the tumor necrosis factor family; BAFF‐R, BAFF receptor; BCMA, B cell maturation antigen; CI, confidence interval; OR, odds ratio; SLE, systemic lupus erythematosus; TACI, transmembrane activator and cyclophilin ligand interactor

a Encompasses mild, moderate and severe flares.

### Serum soluble BAFF receptors and SLE clinical parameters

Serum sBCMA was significantly associated with the presence of anti‐dsDNA in univariable analysis (ratio of GM 1.12; 95% CI 1, 1.26; *P *=* *0.05). Serum sBCMA was not associated with overall or organ‐specific disease activity, flare or organ damage (Table**** 3). Longitudinal analysis revealed no significant association between baseline serum sBCMA and clinical parameters over time (Table 5).

Univariable analysis revealed that increased serum sBAFF‐R was associated with Asian ethnicity (ratio of GM 1.42; 95% CI, 1.01, 2.02; *P *=* *0.04) and with serological SLE Disease Activity Index 2000 (SLEDAI‐2K) (ratio of GM 1.35; 95% CI 1.02, 1.78; *P *=* *0.04). However, these associations did not remain significant after adjusting for age (ethnicity: adjusted ratio of GM 1.27; 95% CI 0.87, 1.84; *P *=* *0.22; serological SLEDAI‐2K: adjusted ratio of GM 1.2; 95% CI 0.88, 1.62; *P *=* *0.25). As opposed to BAFF, serum sBAFF‐R was significantly higher in patients receiving hydroxychloroquine (Table 3). No significant association was found between serum sBAFF‐R and any other SLE clinical parameters in cross‐sectional or in longitudinal analyses (Tables 3, 5).

No significant association was found between serum sTACI and any demographic or clinical parameters in cross‐sectional or in longitudinal analyses (Tables 3, 5).

## Discussion

While BAFF is a well‐established therapeutic target in SLE, better understanding of how its expression impacts on disease activity is needed. One possible avenue is the relationship between levels of BAFF and of the soluble forms of the three receptors for BAFF. This study aimed to evaluate the presence of soluble BAFF receptors in serum from a well‐defined SLE cohort and to characterise clinical associations.

Here, we showed the presence of soluble forms of the three BAFF receptors in all serum samples from SLE patients. The presence of sBAFF‐R in human SLE sera has not previously been described. The present study confirmed the presence of both sTACI and sBCMA in sera from SLE patients, in a larger and better‐defined cohort than previously reported.^7^, ^8^ Both serum sTACI and sBCMA, but not sBAFF‐R, were significantly higher in SLE than in HC. Serum concentrations of BAFF were also increased in SLE compared to HC, but this association was attenuated after adjusting for age and ethnicity. Higher serum sTACI and sBCMA concentrations were previously reported in SLE.^2^, ^7^, ^8^ It is noteworthy that no significant association was found between serum BAFF and any of its soluble receptors in SLE in the present study. This is in contrast with the sole previously published study on sBCMA in SLE, which reported a positive correlation between serum BAFF and sBCMA.^8^ Whether the detected levels of BAFF and its soluble receptors in this study include complexed forms with their respective cognate soluble receptors/ligands is not known. Future research is required to identify complexed and free forms of these proteins.

We report that increased serum BAFF concentrations were associated with organ damage and flare of disease, in line with some published studies.^15^, ^16^, ^17^ We also report an association between serum BAFF concentrations and the presence of organ damage at follow‐up visits, in line with a previous report.^17^ Unexpectedly, these cross‐sectional and longitudinal associations of serum BAFF with flare and organ damage were independent of the presence of its three soluble receptors.

Laurent *et al*. recently reported that serum sBCMA was positively correlated with SLEDAI in a cohort of 39 SLE patients.^8^ They also showed that sBCMA acted as a decoy only for APRIL.^8^ However, we did not find any statistically significant association between sBCMA and disease activity. Moreover, we did not find any association between sBCMA and clinical phenotypes, flare or organ damage. Increased serum sBCMA was significantly associated with anti‐dsDNA levels, in line with the trend reported by Laurent *et al*.^8^ Similarly, in contrast to the positive correlation reported between serum sTACI and SLEDAI in untreated SLE,^7^ we did not find an association between sTACI and disease activity or any clinical parameters. Discrepancies between our data and these studies may arise from difference in study population with potential difference in phenotypic subsets studied and/or treatment received, as well as in analysis methods.

This work constitutes the first study to show the presence of sBAFF‐R in sera from SLE. We observed a significant association between increased serum sBAFF‐R and active serological SLEDAI‐2K; however, this was attenuated after adjusting for age. Serum sBAFF‐R was not associated with any other SLE clinical parameters in cross‐sectional or longitudinal analyses. Interestingly, a negative correlation between serum BAFF and sBAFF‐R was detected in HC samples, but was not observed in SLE, potentially suggesting a loss of co‐regulation of these balancing forces in favor of unopposed BAFF action in SLE; this is consistent with the failure to observed increased sBAFF‐R in SLE despite other BAFF receptors being elevated. Further research in larger studies would help examine this hypothesis, as well as associations between soluble BAFF receptors and phenotypic SLE subsets under‐represented in the present study, such as neurological and musculoskeletal subsets.

SLE is a multifactorial disease, where genetic factors are acknowledged to play an important role in disease pathogenesis.^18^ Consistent with this, SLE is reported of a higher prevalence and more severe in different ethnicities, including Asians and Indigenous Australians, than in Caucasians, even when studied at the same centre.^2^, ^19^ Serum BAFF levels, and the relationship of these with disease activity, have been reported to be different between African American and White American SLE patients.^2^ Here, no significant difference in serum BAFF was found between Asian and Caucasian patients, in line with our previously published work^20^; the same was true for serum sTACI and sBCMA. Asian SLE patients displayed significantly higher serum sBAFF‐R levels than Caucasians, but this association was not confirmed after adjusting for age. Future larger multiethnic studies evaluating whether ethnicity might influence serum sBAFF‐R levels in SLE patients would be of interest.

This study has identifiable limitations. Firstly, recruitment was monocentric. However, the data draw on a very well‐characterised longitudinally followed cohort of SLE patients. Secondly, the HC cohort was of modest size and not age‐matched to the SLE cohort; multivariable regression analysis, however, enabled adjustment for this demographic variable. Finally, numbers of patients with specific active SLE organ manifestations were small in some subsets, such as neurological SLE, precluding meaningful statistical analysis.

In conclusion, we report the presence of the three soluble BAFF receptors in serum from SLE patients. Serum BAFF, sBCMA and sTACI were significantly higher in SLE than in HC. In contrast, serum sBAFF‐R was not elevated in SLE, and the negative correlation between BAFF and sBAFF‐R in HC was not observed in SLE. Serum BAFF was associated with flare of disease and organ damage accrual, independent of the presence of its three soluble cognate receptors, which did not vary strongly with disease activity. These data provide insight into the role of the BAFF system in SLE, and show that all BAFF receptors are detectable in this disease, but leave open the need for larger studies of the functional relations between BAFF and its receptors in SLE.

## Methods

### Patients and clinical assessments

Adult SLE patients fulfilling the 1997 American College of Rheumatology (ACR) revised criteria for SLE classification^21^ were enrolled from the Lupus Clinic at Monash Medical Centre (Clayton, Victoria, Australia) between December 2009 and July 2014. Patients were not receiving anti‐BAFF, anti‐CD20 or anti‐CD22 drugs within 12 months of sample collection. Demographic and clinical data were recorded prospectively, including date of birth, gender, ethnicity, disease duration, disease activity and treatment. Overall SLE disease activity was assessed using the SLEDAI‐2K,^22^ and SLE was defined as active (SLEDAI‐2K > 4) or inactive (SLEDAI‐2K ≤ 4), as previously described.^23^ The 24 items of the SLEDAI‐2K were individually scored, followed by grouping them into nine organ‐specific domains (e.g. neurological, mucocutaneous, renal) in order to assess organ‐specific disease activity. For example, renal disease activity was measured by adding the four renal components of the SLEDAI‐2K: proteinuria, haematuria, urinary casts and pyuria (renal SLEDAI‐2K)^24^; active renal disease was defined as a renal SLEDAI‐2K score > 0. The AMS was calculated as the SLEDAI‐2K area under the curve divided by the time observed, as previously described.^25^ Active disease over time was defined as AMS > 4, as previously described.^26^ Flare was defined according to SLE Flare Index.^27^ Damage was recorded using the Systemic Lupus International Collaborating Clinics (SLICC) SLE Damage Index (SDI), as previously described.^20^, ^25^ Clinical data were collected for a median of two years following the serum sampling. Healthy individuals were enrolled as a HC group. All individuals gave written informed consent. This study was approved by the Monash Health Human Research Ethics Committee. The study was carried out in accordance with the *National Statement of Ethical Conduct in Human Research (2007)*.

### Collection of human biological samples

Blood samples were collected by venepuncture at the time of routine clinical assessment. Serum was isolated using serum‐separating blood collection tube, and stored at −80°C until further use, as previously described.^28^

### Serum cytokine and soluble receptors quantification

Commercial ELISA kits were used to quantify serum BAFF (Quantikine, Cat #SBLYS0B, R&D Systems, Minneapolis, MN, USA) and serum sBAFF‐R (Cat #qy‐e05097, Qayee‐Bio, Shanghai, China), following the manufacturer's protocols. A commercial Luminex screening assay kit (polystyrene beads; Cat #LXSAH, R&D Systems) was used to quantify serum sBCMA and sTACI concentrations, using a Bio‐Rad Bio‐Plex 200 system, following the manufacturer's protocol. Serum sBAFF‐R concentrations were not quantified in 6 samples over the 17 HC serum samples tested for BAFF and its soluble receptors.

### Statistical analysis

All statistical analyses were performed using Stata version 14.2 (StataCorp, College Station, Texas, USA). Normally distributed data were presented as mean (standard deviation) (SD). Non‐normally distributed data were summarised as median and IQR. Spearman's correlation test was used to examine the correlations between BAFF and its soluble receptors (sBAFF‐R, sBMCA and sTACI). Categorical variables were analysed by Pearson's chi‐squared test or Fisher's exact test when appropriate. The Mann–Whitney *U*‐test was used to assess differences in non‐normal continuous variables between the SLE and HC groups.

Cytokine and soluble receptors concentrations were positively skewed in distribution and were therefore log_10_‐transformed before being used as outcomes in linear regression analyses. Bootstrap methods were incorporated to derive robust standard errors when data did not resemble perfect normal distribution even after log_10_ transformation. Potential confounders were tested for inclusion in multivariable regression models, including demographic and treatment data. All three serum soluble BAFF receptors were tested as potential confounders for association between serum BAFF and clinical parameters, and vice versa. In longitudinal analysis, serum concentrations were categorised into binary variables, using their medians as a cut‐off (≤ median = low, > median = high), to be used as exposures in a logistic regression model. Non‐intermediary variables associated with both primary exposure and outcome variables were included in the multivariable analyses as potential confounders. A *P*‐value of < 0.1 in univariable analysis was used as threshold to select potential confounders for multivariable regression models. A *P*‐value of < 0.05 was considered statistically significant.

## Data Availability

Reasonable requests to view the data set used in this manuscript can be made in writing to Dr Fabien Vincent (fabien.vincent@monash.edu).

## References

[1] Lisnevskaia L , Murphy G , Isenberg D . Systemic lupus erythematosus. Lancet 2014; 384: 1878–1888. 24881804 10.1016/S0140-6736(14)60128-8

[2] Vincent FB , Morand EF , Schneider P *et al* The BAFF/APRIL system in SLE pathogenesis. Nat Rev Rheumatol 2014; 10: 365–373. 24614588 10.1038/nrrheum.2014.33

[3] Mackay F , Woodcock SA , Lawton P *et al* Mice transgenic for BAFF develop lymphocytic disorders along with autoimmune manifestations. J Exp Med 1999; 190: 1697–1710. 10587360 10.1084/jem.190.11.1697 PMC2195729

[4] Vincent FB , Saulep‐Easton D , Figgett WA *et al* The BAFF/APRIL system: emerging functions beyond B cell biology and autoimmunity. Cytokine Growth Factor Rev 2013; 24: 203–215. 23684423 10.1016/j.cytogfr.2013.04.003 PMC7108297

[5] Navarra SV , Guzman RM , Gallacher AE *et al* Efficacy and safety of belimumab in patients with active systemic lupus erythematosus: a randomised, placebo‐controlled, phase 3 trial. Lancet 2011; 377: 721–731. 21296403 10.1016/S0140-6736(10)61354-2

[6] Furie R , Petri M , Zamani O *et al* A phase III, randomized, placebo‐controlled study of belimumab, a monoclonal antibody that inhibits B lymphocyte stimulator, in patients with systemic lupus erythematosus. Arthritis Rheum 2011; 63: 3918–3930. 22127708 10.1002/art.30613 PMC5007058

[7] Hoffmann FS , Kuhn PH , Laurent SA *et al* The immunoregulator soluble TACI is released by ADAM10 and reflects B cell activation in autoimmunity. J Immunol 2015; 194: 542–552. 25505277 10.4049/jimmunol.1402070 PMC4282951

[8] Laurent SA , Hoffmann FS , Kuhn PH *et al* gamma‐Secretase directly sheds the survival receptor BCMA from plasma cells. Nat Commun 2015; 6: 7333. 26065893 10.1038/ncomms8333 PMC4490565

[9] Schuh E , Musumeci A , Thaler FS *et al* Human plasmacytoid dendritic cells display and shed B cell maturation antigen upon TLR engagement. J Immunol 2017; 198: 3081–3088. 28283566 10.4049/jimmunol.1601746

[10] Deng BP , Zhang Y , Wang QJ *et al* Soluble BAFF‐R produced by decidual stromal cells plays an inhibitory role in monocytes and macrophages. Reprod Biomed 2012; 24: 654–663. 10.1016/j.rbmo.2012.02.024 22503273

[11] Rodriguez‐Carrio J , Alperi‐Lopez M , Lopez P *et al* Profiling of B‐cell factors and their decoy receptors in rheumatoid arthritis: association with clinical features and treatment outcomes. Front Immunol 2018; 9: 2351. 30369929 10.3389/fimmu.2018.02351 PMC6194314

[12] Thompson SA , Jones JL , Cox AL *et al* B‐cell reconstitution and BAFF after alemtuzumab (Campath‐1H) treatment of multiple sclerosis. J Clin Immunol 2010; 30: 99–105. 19763798 10.1007/s10875-009-9327-3

[13] Sanchez E , Li M , Kitto A *et al* Serum B‐cell maturation antigen is elevated in multiple myeloma and correlates with disease status and survival. Br J Haematol 2012; 158: 727–738. 22804669 10.1111/j.1365-2141.2012.09241.x

[14] Kyrtsonis M‐C , Sarris K , Koulieris E *et al* Serum soluble TACI, a BLyS receptor, is a powerful prognostic marker of outcome in chronic lymphocytic leukemia. Biomed Res Int 2014; 2014: 1–5. 10.1155/2014/159632 PMC4138780 25162001

[15] Petri MA , van Vollenhoven RF , Buyon J *et al* Baseline predictors of systemic lupus erythematosus flares: data from the combined placebo groups in the phase III belimumab trials. Arthritis Rheum 2013; 65: 2143–2153. 23754628 10.1002/art.37995

[16] Carter LM , Isenberg DA , Ehrenstein MR . Elevated serum BAFF levels are associated with rising anti‐double‐stranded DNA antibody levels and disease flare following B cell depletion therapy in systemic lupus erythematosus. Arthritis Rheum 2013; 65: 2672–2679. 23839909 10.1002/art.38074

[17] McCarthy EM , Lee RZ , Ni Gabhann J *et al* Elevated B lymphocyte stimulator levels are associated with increased damage in an Irish systemic lupus erythematosus cohort. Rheumatology (Oxford) 2013; 52: 1279–1284. 23479724 10.1093/rheumatology/ket120

[18] Wahren‐Herlenius M , Dorner T . Immunopathogenic mechanisms of systemic autoimmune disease. Lancet 2013; 382: 819–831. 23993191 10.1016/S0140-6736(13)60954-X

[19] Vincent FB , Bourke P , Morand EF *et al* Focus on systemic lupus erythematosus in indigenous Australians: towards a better understanding of autoimmune diseases. Intern Med J 2013; 43: 227–234. 23176380 10.1111/imj.12039

[20] Vincent FB , Northcott M , Hoi A *et al* Association of serum B cell activating factor from the tumour necrosis factor family (BAFF) and a proliferation‐inducing ligand (APRIL) with central nervous system and renal disease in systemic lupus erythematosus. Lupus 2013; 22: 873–884. 23846230 10.1177/0961203313496302

[21] Hochberg MC . Updating the American College of Rheumatology revised criteria for the classification of systemic lupus erythematosus. Arthritis Rheum 1997; 40: 1725. 10.1002/art.1780400928 9324032

[22] Gladman DD , Ibanez D , Urowitz MB . Systemic lupus erythematosus disease activity index 2000. J Rheumatol 2002; 29: 288–291. 11838846

[23] Mende R , Vincent FB , Kandane‐Rathnayake R *et al* Analysis of serum interleukin (IL)‐1β and IL‐18 in systemic lupus erythematosus. Front Immunol 2018; 9: 1250. 29930551 10.3389/fimmu.2018.01250 PMC5999794

[24] Pitashny M , Schwartz N , Qing X *et al* Urinary lipocalin‐2 is associated with renal disease activity in human lupus nephritis. Arthritis Rheum 2007; 56: 1894–1903. 17530720 10.1002/art.22594

[25] Vincent FB , Northcott M , Hoi A *et al* Clinical associations of serum interleukin‐17 in systemic lupus erythematosus. Arthritis Res Ther 2013; 15: R97. 23968496 10.1186/ar4277 PMC3979031

[26] Godsell J , Rudloff I , Kandane‐Rathnayake R *et al* Clinical associations of IL‐10 and IL‐37 in systemic lupus erythematosus. Sci Rep 2016; 6: 34604. 27708376 10.1038/srep34604 PMC5052569

[27] Petri M , Kim MY , Kalunian KC *et al* Combined oral contraceptives in women with systemic lupus erythematosus. N Engl J Med 2005; 353: 2550–2558. 16354891 10.1056/NEJMoa051135

[28] Vincent FB , Nim HT , Lee JPW *et al* Effect of storage duration on cytokine stability in human serum and plasma. Cytokine 2019; 113: 453–457. 29909979 10.1016/j.cyto.2018.06.009

