# Supplementary material for: Analysis of serum B cell‐activating factor from the tumor necrosis factor family (BAFF) and its soluble receptors in systemic lupus erythematosus
Source: Clin Transl Immunology. 2019 Apr 21;8(4):e01047. doi: 10.1002/cti2.1047 (PMC6475618; doi:10.1002/cti2.1047)
Supplement: Supplementary file 1 [file CTI2-8-e01047-s001.docx]

**Supplementary Table 1. Univariable and multivariable associations of serum BAFF in SLE compared to HC.**

|  | **Serum BAFF levels (pg mL^-1^)** | | | | | **Serum BAFF levels (pg mL^-1^)** | | | | |
| --- | --- | --- | --- | --- | --- | --- | --- | --- | --- | --- |
|  | **derived from univariable linear regression analyses** | | | | | **derived from multivariable linear regression analyses** | | | | |
| ***Exposures*** |  |  | **Regression coef.** | **(95% CI)** | ***P*-value** |  |  | **Regression coef.** | **(95% CI)** | ***P*-value** |
| **Age** |  |  | 1.004 | (0.99, 1.01) | 0.23 |  |  | 1.00 | (1.00, 1.01) | 0.46 |
|  |  |  |  |  |  |  |  |  |  |  |
|  | **GM** | **(95% CI)** | **Ratio of GM** | **(95% CI)** | ***P*-value** | **GM** | **(95% CI)** | **Ratio of GM** | **(95% CI)** | ***P*-value** |
| **Disease** |  |  |  |  |  |  |  |  |  |  |
| HC | 1021 | (813, 1281) | 1.00 |  |  | 1032 | (812, 1313) | 1.00 |  |  |
| SLE | 1298 | (1174, 1435) | 1.27 | (0.99, 1.63) | 0.06 | 1295 | (1169, 1434) | 1.25 | (0.96, 1.64) | 0.09 |
| **Ethnicity** |  |  |  |  |  |  |  |  |  |  |
| Non-Asian | 1253 | (1095, 1435) | 1.00 |  |  | 1265 | (1103, 1452) | 1.00 |  |  |
| Asian | 1243 | (1091, 1415) | 0.99 | (0.82, 1.2) | 0.93 | 1232 | (1079, 1406) | 0.97 | (0.8, 1.18) | 0.78 |

95% CI: 95% Confidence Interval; BAFF: B cell-activating factor from the tumor necrosis factor family; GM: Geometric mean; HC: healthy control; SLE: systemic lupus erythematosus.
