# Supplementary material for: Analysis of serum B cell‐activating factor from the tumor necrosis factor family (BAFF) and its soluble receptors in systemic lupus erythematosus
Source: Clin Transl Immunology. 2019 Apr 21;8(4):e01047. doi: 10.1002/cti2.1047 (PMC6475618; doi:10.1002/cti2.1047)
Supplement: Supplementary file 2 [file CTI2-8-e01047-s002.docx]

**Supplementary Table 2. Univariable and multivariable associations of serum sBCMA in SLE compared to HC.**

|  | **Serum sBCMA levels (pg mL^-1^)** | | | | | **Serum sBCMA levels (pg mL^-1^)** | | | | |
| --- | --- | --- | --- | --- | --- | --- | --- | --- | --- | --- |
|  | **derived from univariable linear regression analyses** | | | | | **derived from multivariable linear regression analyses** | | | | |
| ***Exposures*** |  |  | **Regression coef.** | **(95% CI)** | ***P*-value** |  |  | **Regression coef.** | **(95% CI)** | ***P*-value** |
| **Age** |  |  | 1.00 | (1.00, 1.01) | 0.03 |  |  | 1.00 | (1.00, 1.01) | 0.26 |
|  |  |  |  |  |  |  |  |  |  |  |
|  | **GM** | **(95% CI)** | **Ratio of GM** | **(95% CI)** | ***P*-value** | **GM** | **(95% CI)** | **Ratio of GM** | **(95% CI)** | ***P*-value** |
| **Disease** |  |  |  |  |  |  |  |  |  |  |
| HC | 12886 | (12013, 13822) | 1.00 |  |  | 13094 | (11997, 14291) | 1.00 |  |  |
| SLE | 18796 | (17880, 19758) | 1.46 | (1.35, 1.58) | <0.01 | 18737 | (17859, 19658) | 1.43 | (1.28, 1.59) | <0.01 |
| **Ethnicity** |  |  |  |  |  |  |  |  |  |  |
| Non-Asian | 17408 | (15827, 19146) | 1.00 |  |  | 17749 | (16627, 18948) | 1.00 |  |  |
| Asian | 17918 | (16953, 18939) | 1.03 | (0.92, 1.15) | 0.62 | 17599 | (16676, 18572) | 0.99 | (0.91, 1.08) | 0.85 |

95% CI: 95% Confidence Interval; BCMA: B cell maturation antigen; GM: Geometric mean; HC: healthy control; SLE: systemic lupus erythematosus.
