# Supplementary material for: Analysis of serum B cell‐activating factor from the tumor necrosis factor family (BAFF) and its soluble receptors in systemic lupus erythematosus
Source: Clin Transl Immunology. 2019 Apr 21;8(4):e01047. doi: 10.1002/cti2.1047 (PMC6475618; doi:10.1002/cti2.1047)
Supplement: Supplementary file 3 [file CTI2-8-e01047-s003.docx]

**Supplementary Table 3. Univariable and multivariable associations of serum sTACI in SLE compared to HC.**

|  | **Serum sTACI levels (pg mL^-1^)** | | | | | **Serum sTACI levels (pg mL^-1^)** | | | | |
| --- | --- | --- | --- | --- | --- | --- | --- | --- | --- | --- |
|  | **derived from univariable linear regression analyses** | | | | | **derived from multivariable linear regression analyses** | | | | |
| ***Exposures*** |  |  | **Regression coef.** | **(95% CI)** | ***P*-value** |  |  | **Regression coef.** | **(95% CI)** | ***P*-value** |
| **Age** |  |  | 1.00 | (0.99, 1.01) | 0.78 |  |  | 1.00 | (0.99, 1.01) | 0.94 |
|  |  |  |  |  |  |  |  |  |  |  |
|  | **GM** | **(95% CI)** | **Ratio of GM** | **(95% CI)** | ***P*-value** | **GM** | **(95% CI)** | **Ratio of GM** | **(95% CI)** | ***P*-value** |
| **Disease** |  |  |  |  |  |  |  |  |  |  |
| HC | 19 | (15, 25) | 1.00 |  |  | 20 | (16, 25) | 1.00 |  |  |
| SLE | 29 | (26, 32) | 1.47 | (1.11, 1.94) | <0.01 | 29 | (25, 32) | 1.45 | (1.11, 1.91) | <0.01 |
| **Ethnicity** |  |  |  |  |  |  |  |  |  |  |
| Non-Asian | 25 | (21, 31) | 1.00 |  |  | 26 | (22, 32) | 1.00 |  |  |
| Asian | 28 | (25, 32) | 1.11 | (0.86, 1.45) | 0.46 | 27 | (23, 32) | 1.04 | (0.8, 1.37) | 0.75 |

95% CI: 95% Confidence Interval; GM: Geometric mean; HC: healthy control; SLE: systemic lupus erythematosus; TACI: transmembrane activator and cyclophilin ligand interactor.
